# [Expression of Concern] Hypoxia and macrophages promote glioblastoma invasion by the CCL4-CCR5 axis

**DOI:** 10.3892/or.2025.8989

**Published:** 2025-09-16

**Authors:** Ying Wang, Tao Liu, Ning Yang, Shuo Xu, Xingang Li, Donghai Wang

Oncol Rep 36: 3522–3528, 2016; DOI: 10.3892/or.2016.5171

Following the publication of this paper, it was drawn to the Editor's attention by a concerned reader that the ‘Normoxia / U87’ panel in Fig. 1A appeared to overlap with the ‘CCR5 siRNA’ panel in Fig. 2B; in addition, the ‘Hypoxia / U87-Mφ’ panel in Fig. 1A appeared to overlap with the ‘Control siRNA’ panel in Fig. 2B.

The authors were contacted by the Editorial Office to offer an explanation for these apparent anomalies in the presentation of the data in this paper; however, up to this time, no response from them has been forthcoming. Owing to the fact that the Editorial Office has been made aware of potential issues surrounding the scientific integrity of this paper, we are issuing an Expression of Concern to notify readers of this potential problem while the Editorial Office continues to investigate this matter further.

